# Papillary Glioneuronal Tumour: A Case Report

**DOI:** 10.7759/cureus.4215

**Published:** 2019-03-11

**Authors:** Saulius Rocka, Laura Neverauskiene, Ewell L Nelson, Sigita Burneikiene

**Affiliations:** 1 Neurosurgery, Vilnius University, Vilnius, LTU; 2 Pathology, Patologijos Diagnostika, Vilnius, LTU; 3 Neurosurgery, Boulder Neurosurgical Associates, Boulder, USA; 4 Neurosurgery, Justin Parker Neurological Institute, Boulder, USA

**Keywords:** papillary glioneuronal tumour, case report

## Abstract

Only a few cases of papillary glioneuronal tumour (PGNT) with predominantly focal symptomatology are described in the literature. We report on the clinical, radiological, and histopathological features of PGNT. The intraoperative pathology revealed no tumour in the walls of the cyst, thus surgical resection of the nodule was performed leaving the cyst wall intact. There was no recurrence of tumour at the three-year follow-up, although a long-term follow-up is necessary.

## Introduction

Papillary glioneuronal tumour (PGNT) is an uncommon central nervous system tumour type. The first time this name was used by Komori et al. in 1998 [1], though tumours with similar morphology were described a few years earlier: pseudopapillary neurocytoma with glial differentiation [2] and ganglioneurocytoma [3]. PGNT still was a variant of ganglioglioma in the World Health Organization (WHO) classification of brain tumours published in 2000 but was recognized as a distinct disease entity in 2007 [4]. The tumour has been assigned to a grade I, however, a few aggressive behavior variants have been also reported [5-7]. To our knowledge, about 100 cases of PGNT have been described in the literature, but only a few patients with predominantly focal neurological deficits were reported [8-10]. We report on the clinical, radiological, and histopathological features of an additional example of PGNT. Surgical resection of the tumour was performed leaving the cyst wall intact.

## Case presentation

**Figure 1:**
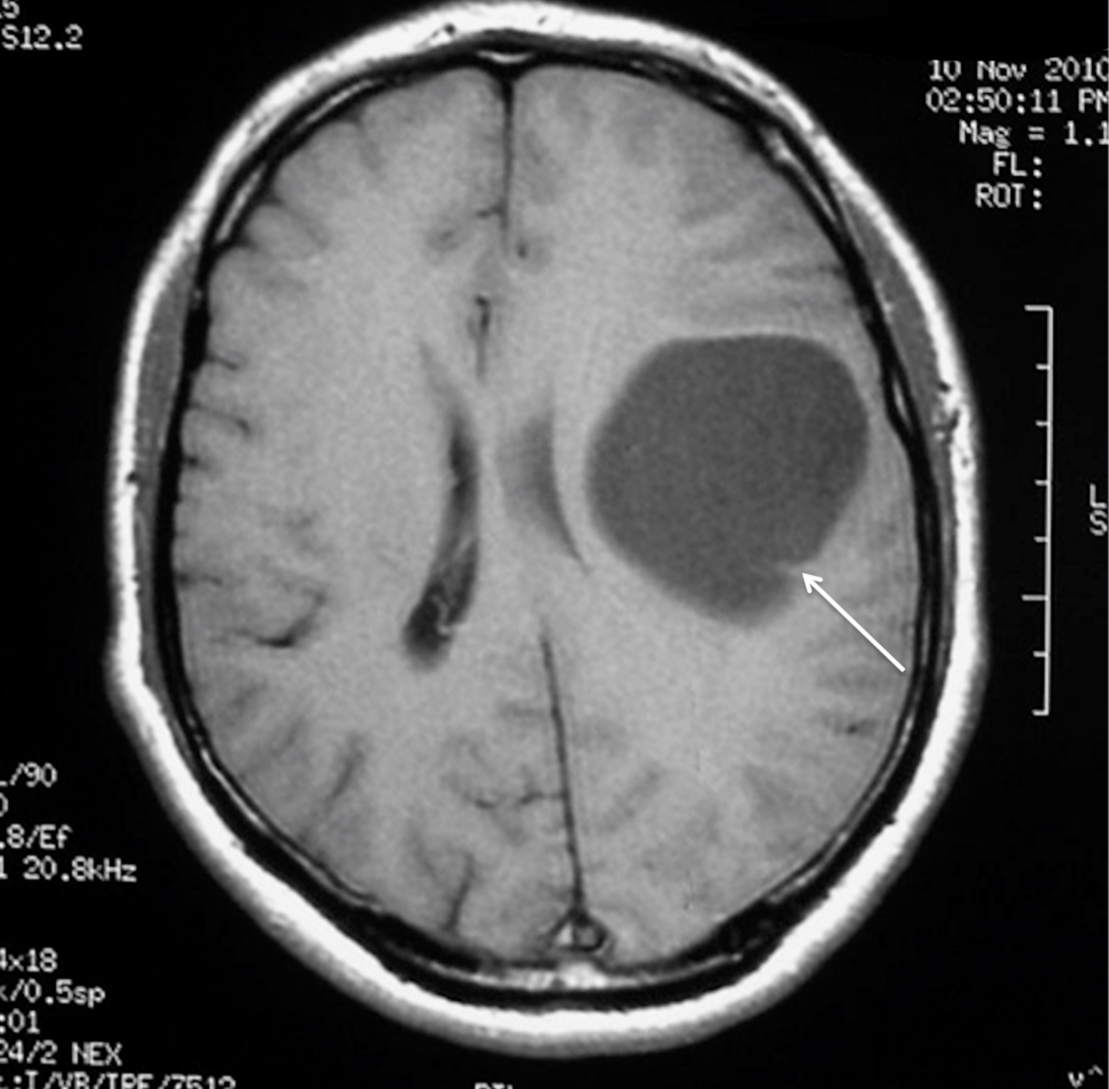
Preoperative MRI without enhancement demonstrating left frontal cystic tumour with septum in the occipital part on the T1WI axial view (arrow). MRI: Magnetic resonance imaging; T1WI: T1-weighted images.

**Figure 2:**
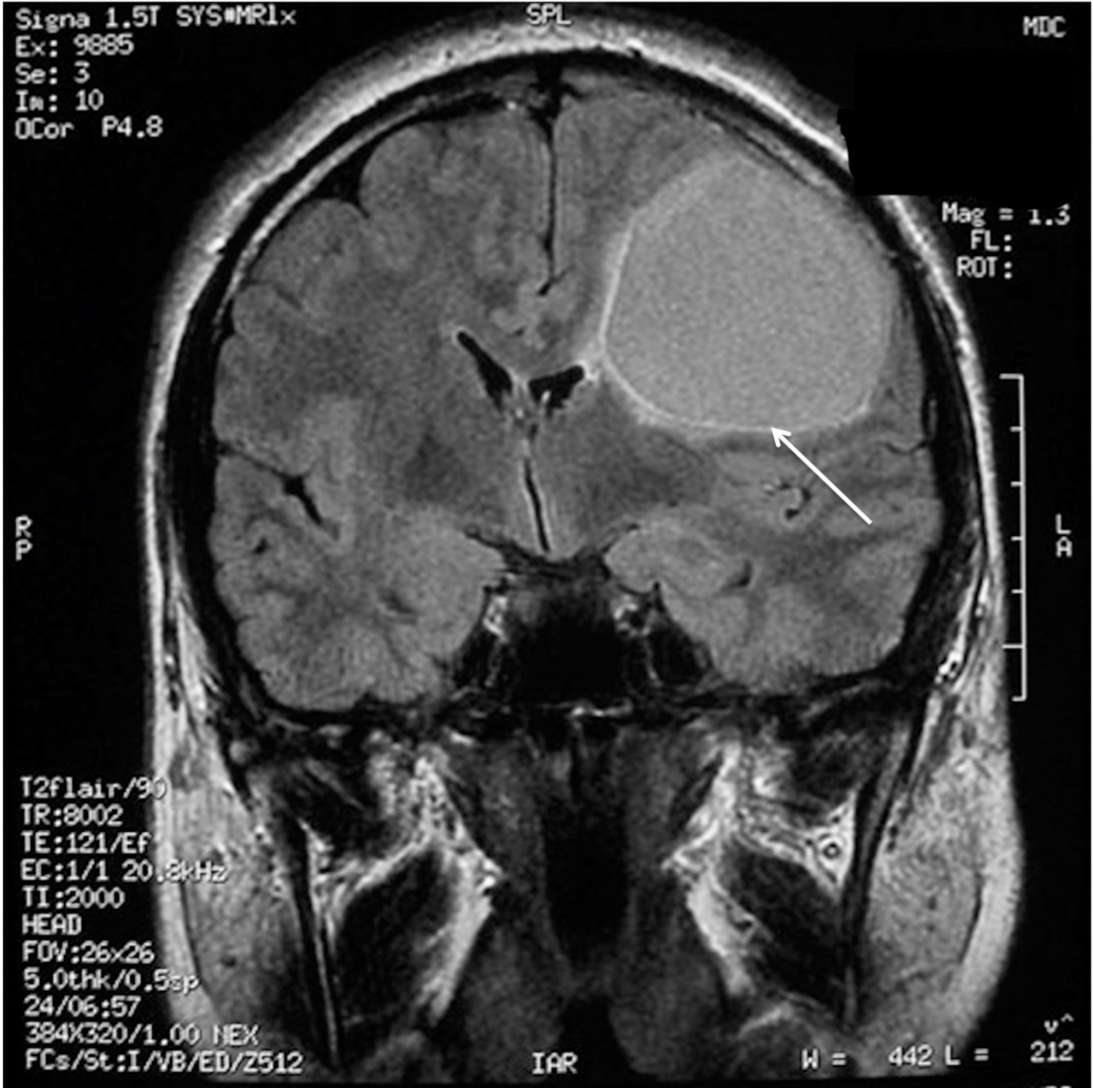
Preoperative MRI without enhancement demonstrating left frontal cystic tumour with hyperintensity of cystic contents on the coronal T2 FLAIR scan (arrow). MRI: Magnetic resonance imaging; FLAIR: Fluid-attenuated inversion recovery.

A 38-year-old, otherwise healthy, right-handed man presented with a two-year progressive history of motor dysphasia and a three-month history of progressive right-hand weakness. The patient also had one short episode of right leg numbness and weakness. A neurological examination showed reflex asymmetry (right > left), hemihypesthesia, hemiparesis (4/5), and positive Babinski sign on the right. Magnetic resonance imaging (MRI) of the brain revealed a 54 x 54 x 52 mm cystic lesion of the left frontal lobe in front of the precentral gyrus with a septum attached to the posterior wall of the cyst. The cyst was hypointense on T1-weighted images (T1WI) (Figure 1) and hyperintense on T2-fluid-attenuated inversion recovery (FLAIR) scans (Figure 2).

**Figure 3:**
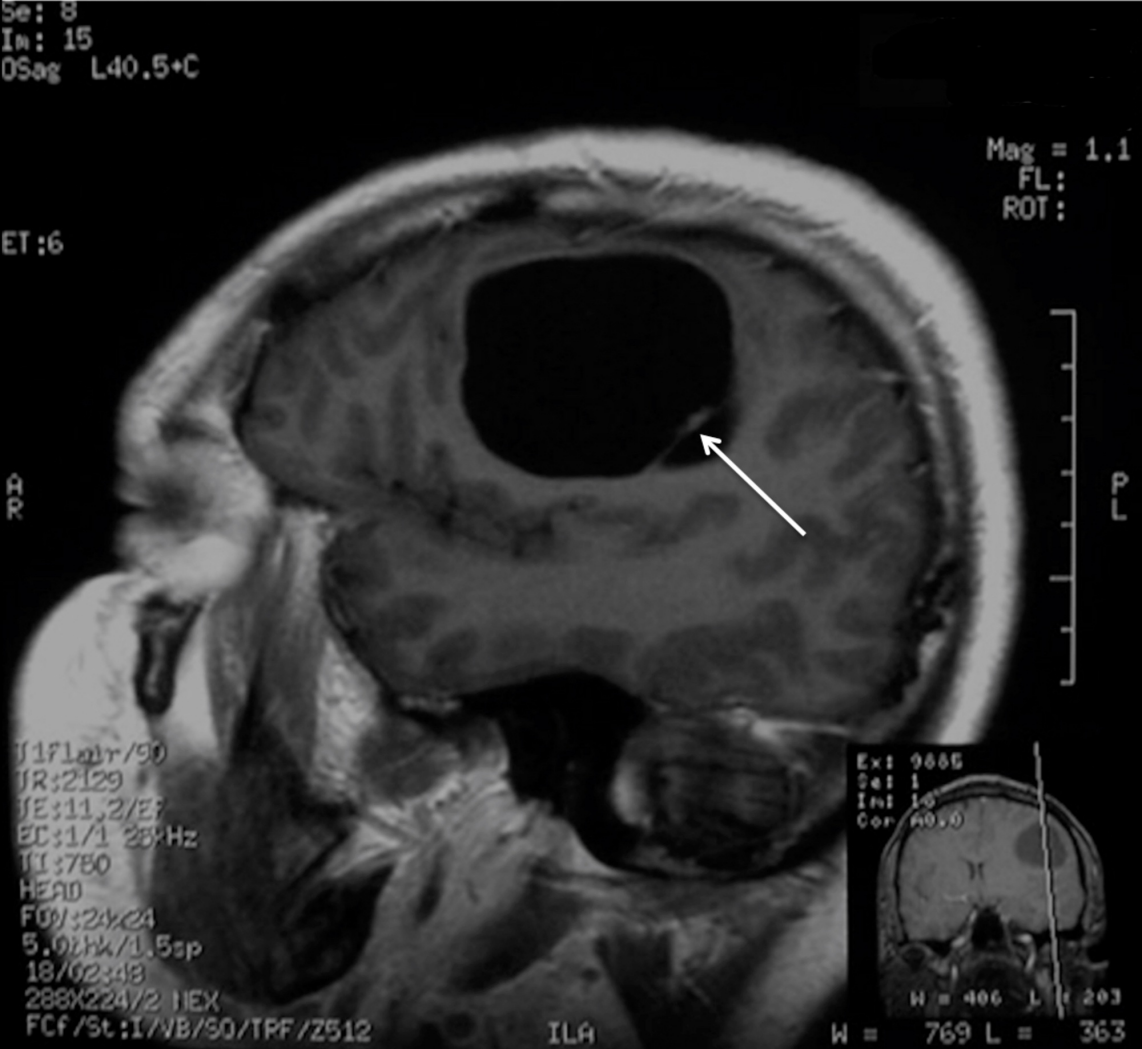
Preoperative MRI demonstrating the contrast-enhancing septum (arrow) in the posterior part of the cyst on the T1WI sagittal view. MRI: Magnetic resonance imaging; T1WI: T1-weighted images.

MRI scans with gadolinium showed a slight contrast accumulation in the cystic walls and homogeneous accumulation in the septum without perifocal edema (Figure 3).

The medial part of the tumour was in close proximity with the left lateral ventricle and subarachnoid spaces were shallow on the left with a 7-mm midline shift present.

**Figure 4:**
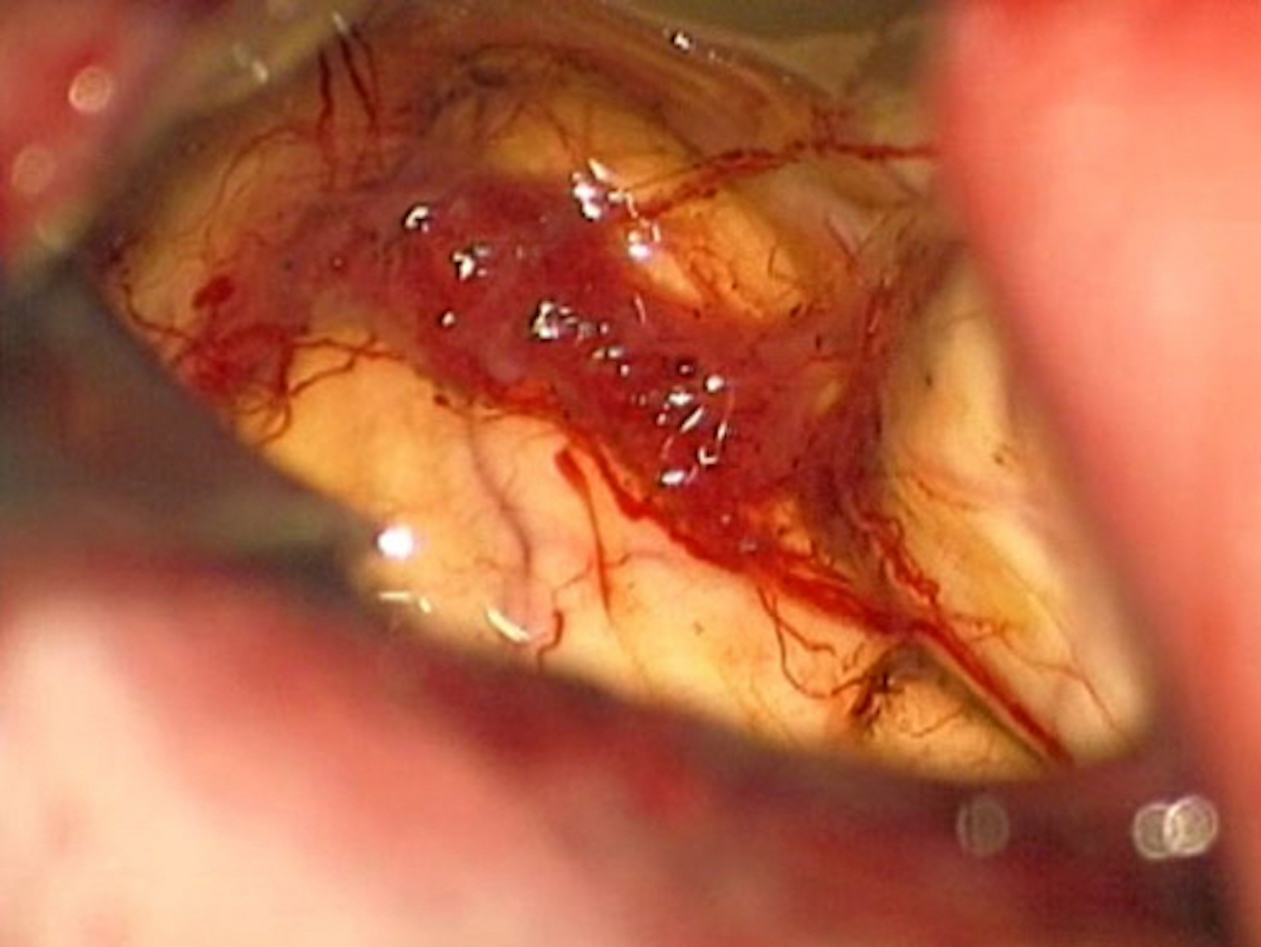
Intraoperative view of the tumour.

The patient was admitted for further examination and treatment. The differential diagnoses included astrocytoma, ganglioglioma, ependymoma, parasitic cyst, and supratentorial cystic hemangioma. The serological examination for echinococcus granulosus was negative and blood examination was normal. Although the final diagnosis was not established, the mass effect was causing clinical deterioration and the patient elected to proceed with surgery for removal of the cystic tumour. Following fronto-temporal craniotomy and a minimal corticotomy, the cystic cavity filled with yellowish fluid was entered. An intraoperative cystic wall biopsy revealed a normal brain tissue and the tumour was found forming the septum on the posterior wall (Figure 4).

The tumour was dissected from the cyst and removed. Taking into account the proximity of the corticospinal tracts and pathology results, the cystic wall was left intact.

**Figure 5:**
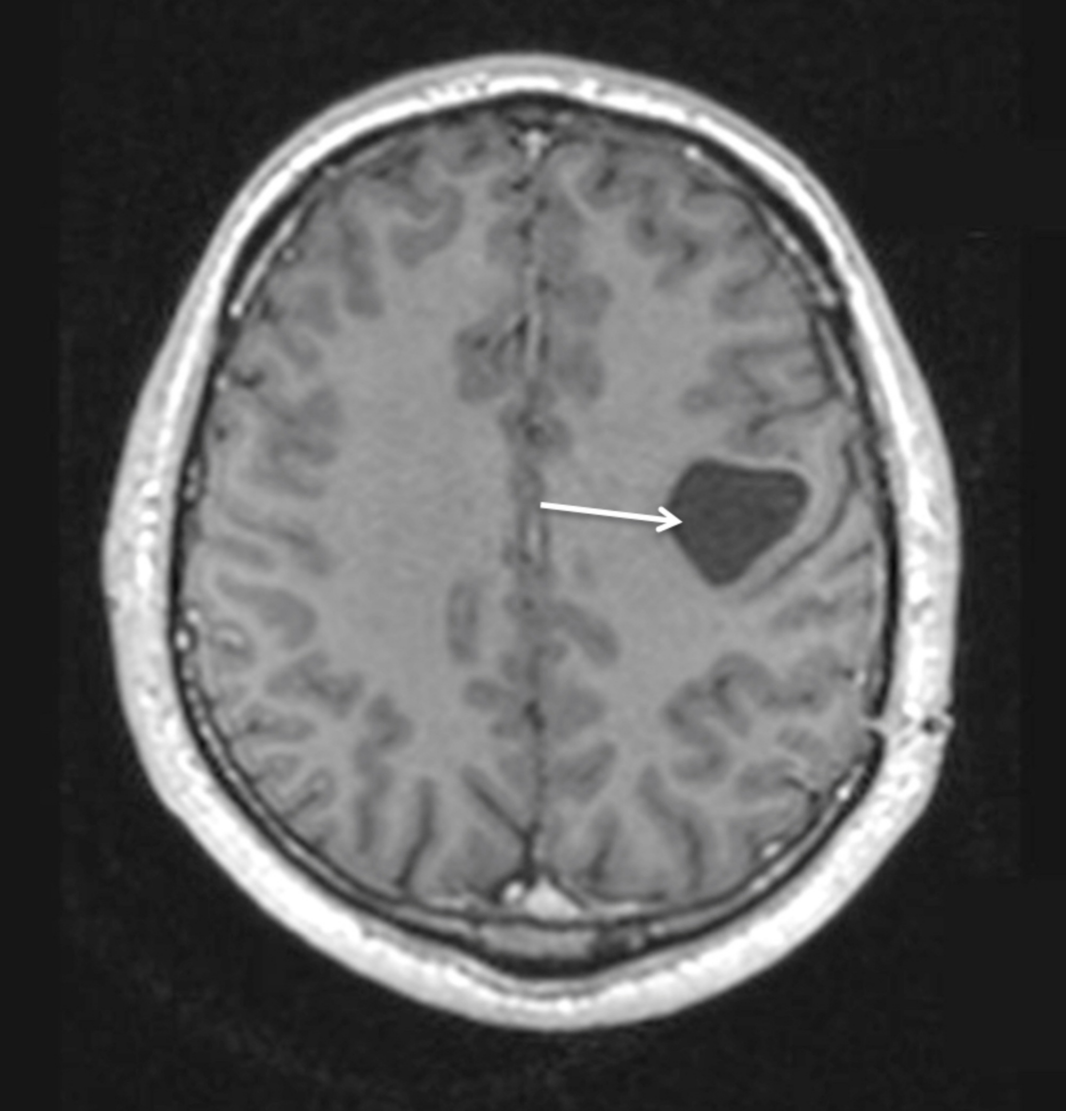
Postoperative MRI with contrast enhancement at eight months follow-up demonstrating the residual cyst without contrast accumulation (arrow), decrease in size, and no mass effect on the T1WI axial view. MRI: Magnetic resonance imaging; T1WI: T1-weighted images.

**Figure 6:**
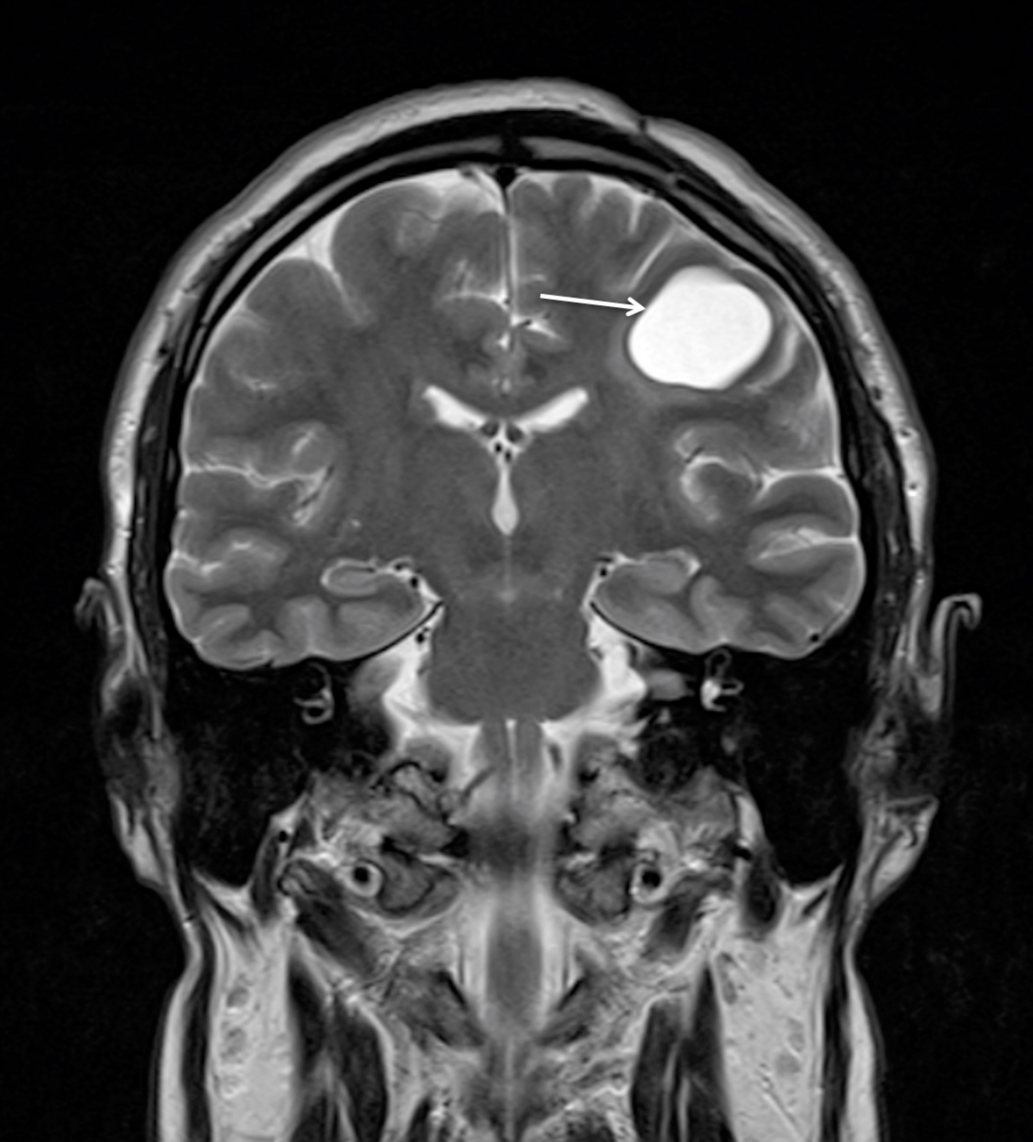
Postoperative MRI with contrast enhancement at eight months follow-up demonstrating the residual cyst (arrow) without contrast accumulation, decrease in size, and no mass effect on the coronal T2WI view. MRI: Magnetic resonance imaging; T2WI: T2-weighted images.

The patient had a non-complicated postoperative course with a full neurological recovery and was discharged on the seven postoperative day. The patient underwent a complete neurological and radiological re-examination eight months after the surgery. He was neurologically intact, MRI showed a residual 2-cm cyst with no enhancement with gadolinium contrast administration (Figures 5, 6).

**Figure 7:**
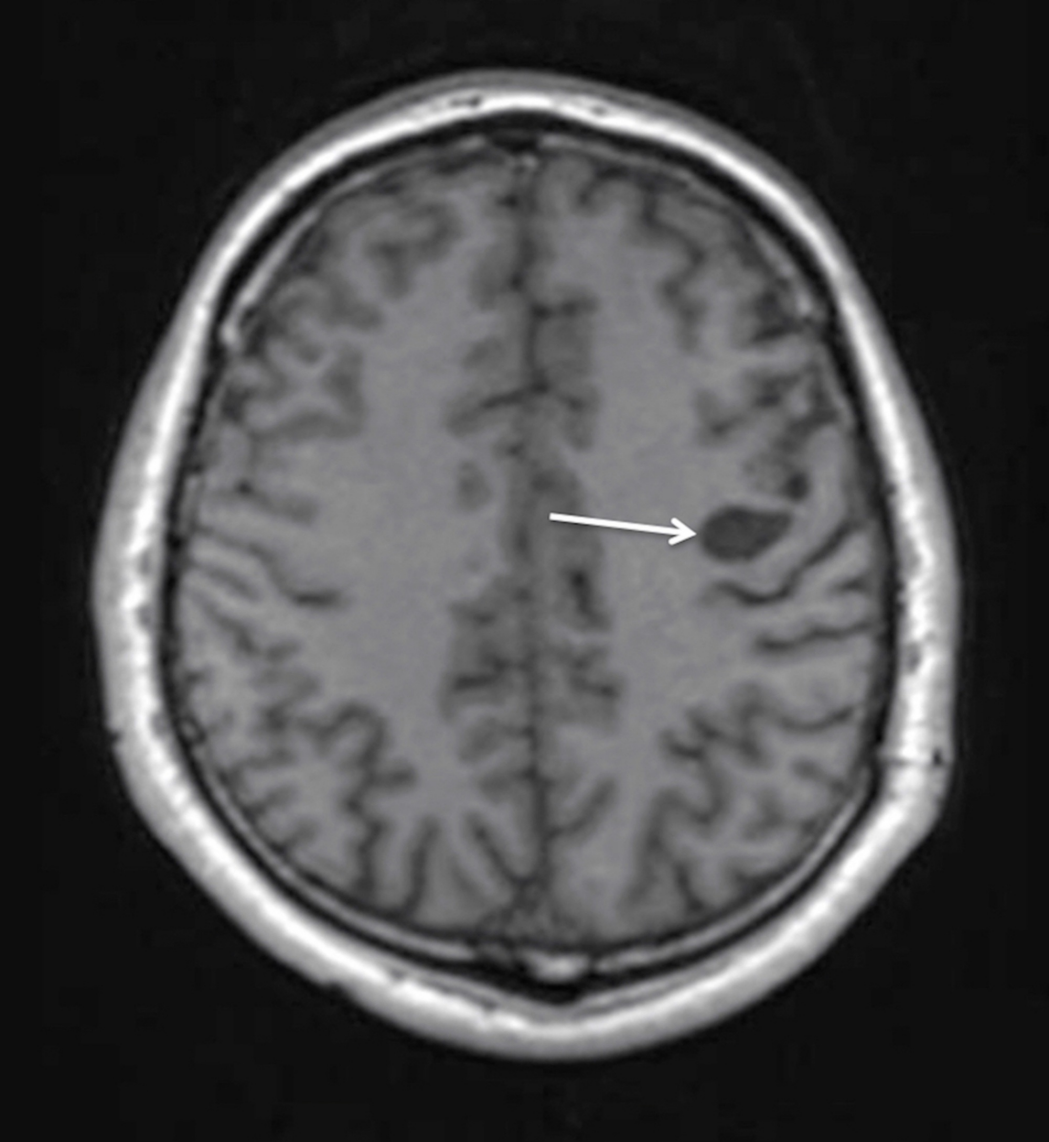
Postoperative MRI with contrast enhancement at two-year follow-up demonstrating the residual cyst (arrow) without contrast accumulation, further decrease in size, and no mass effect on the T1WI axial view. MRI: Magnetic resonance imaging; T1WI: T1-weighted images.

**Figure 8:**
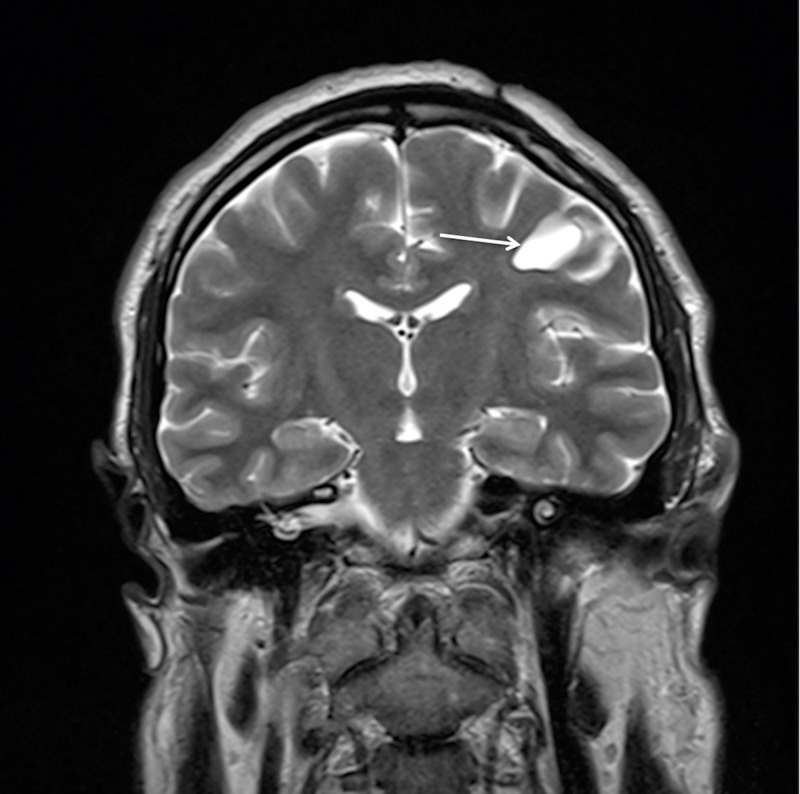
Postoperative MRI with contrast enhancement at two-year follow-up demonstrating the residual cyst (arrow) without contrast accumulation, a further decrease in size, and no mass effect on the T2WI coronal view. MRI: Magnetic resonance imaging; T2WI: T2-weighted images.

He remained symptom-free three years after surgery with a further decrease in the size of the residual cyst on MRI (Figures 7, 8).

Pathology

**Figure 9:**
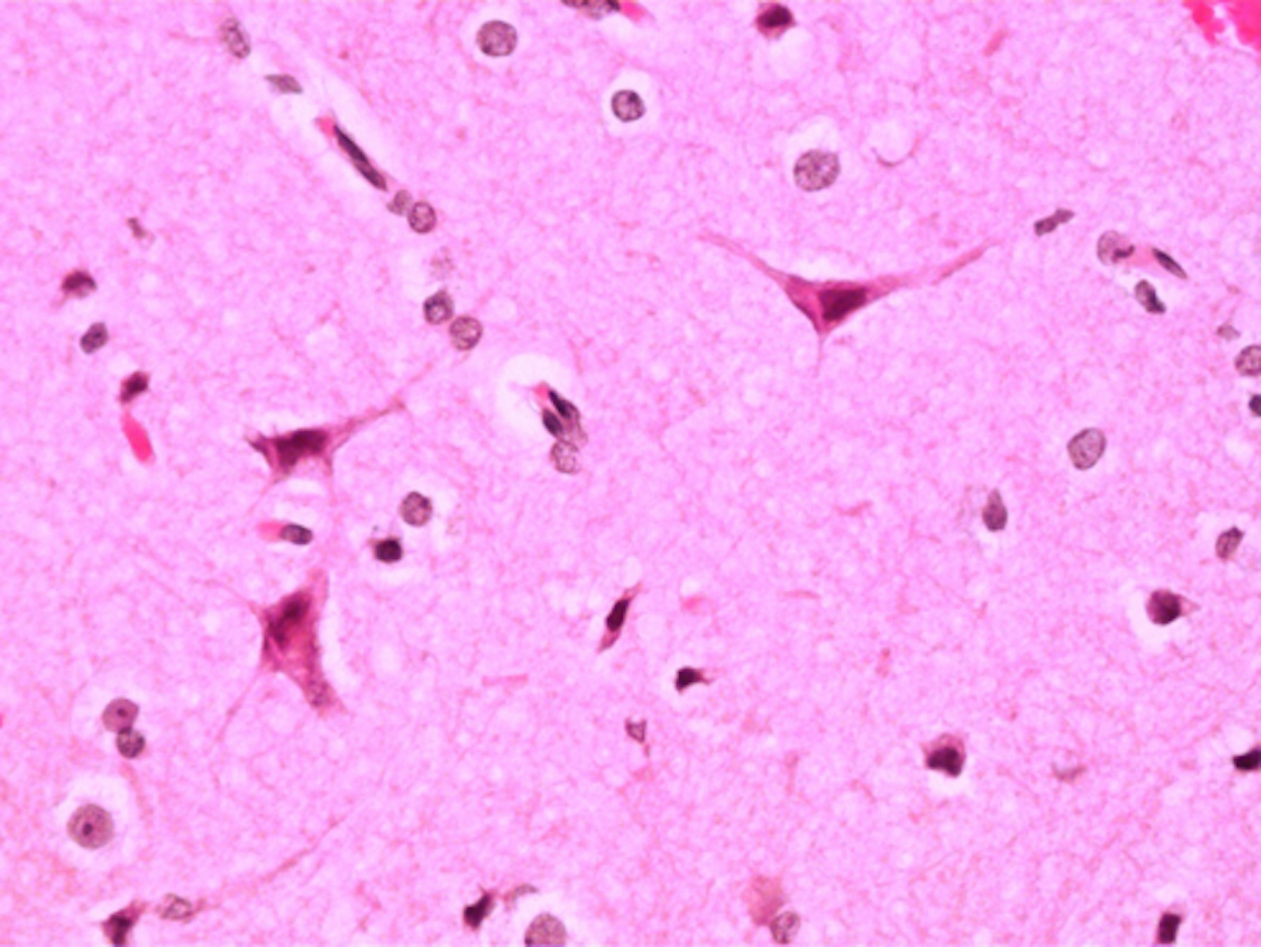
A 40x magnification view of normal brain tissue from the cyst wall.

The intraoperative specimen of the cyst wall was frozen at -15 degrees of Celsius and normal brain tissue was identified on the frozen section slide (Figure 9).

**Figure 10:**
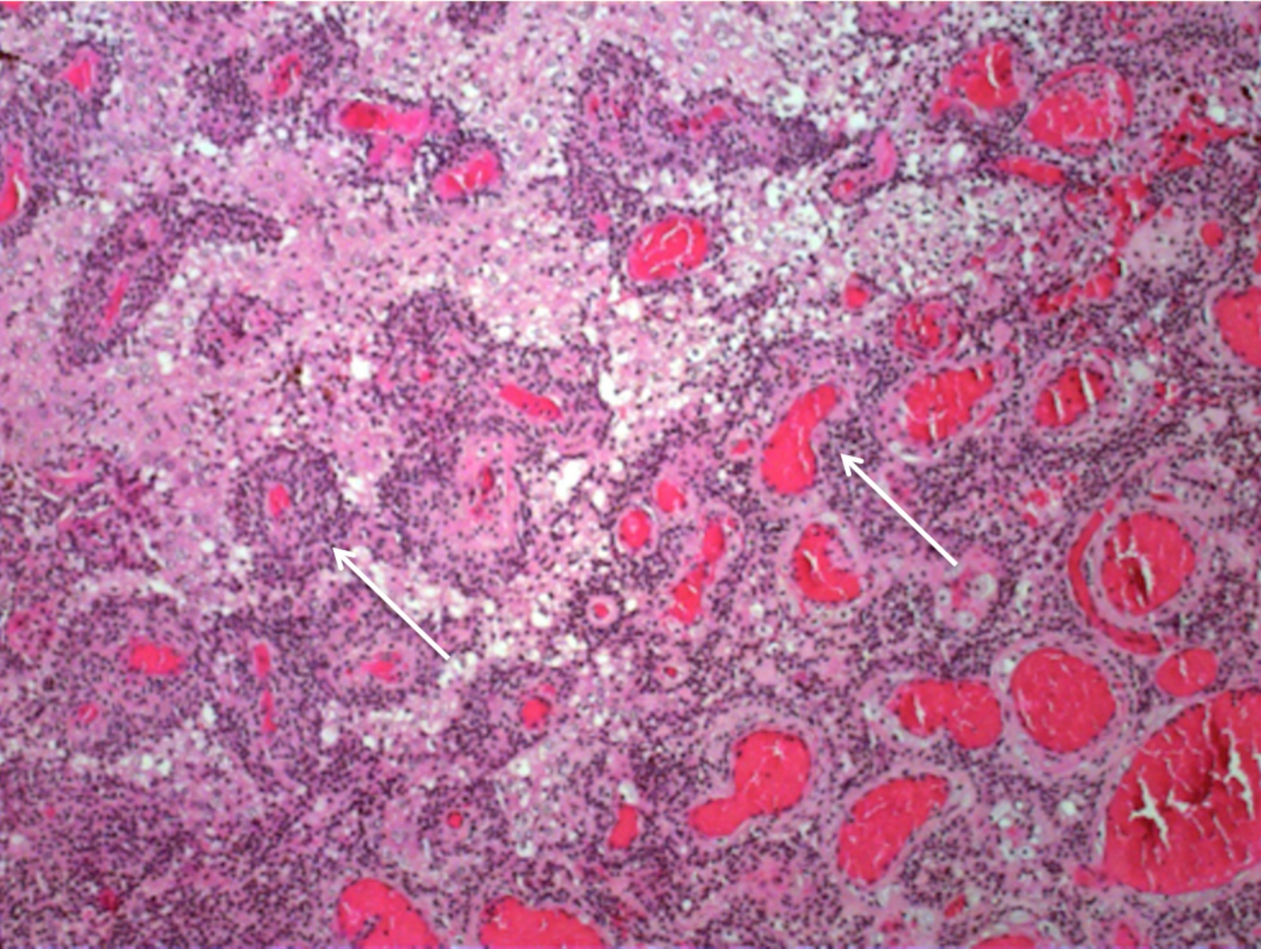
A 40x magnification view of characteristic papillary structures (arrows).

**Figure 11:**
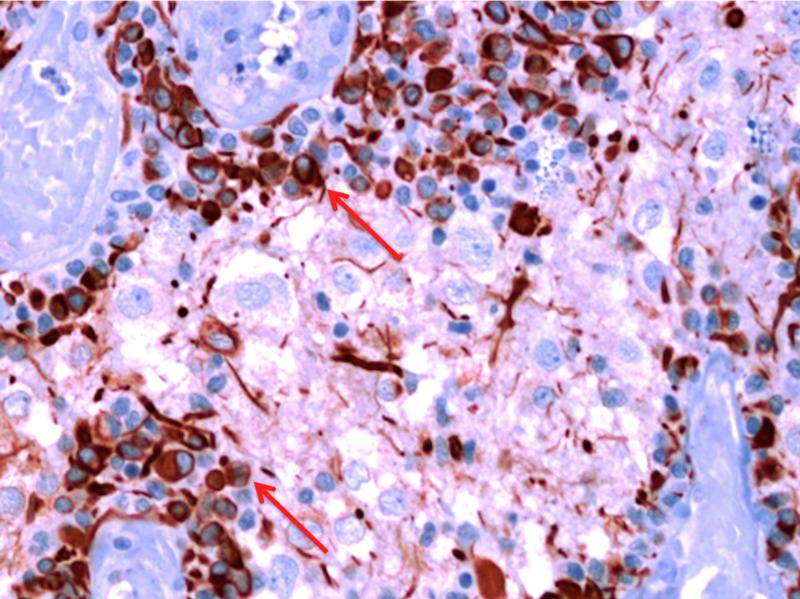
A 40x magnification view of characteristic papillary structures (arrows).

The tumour specimens were fixed in 10% neutral-buffered formalin, processed, and embedded in paraffin, then 3-μm-thick paraffin sections were stained haematoxylin and eosin (H&E) and 5-μm sections were stained immunohistochemically for glial fibrillary acidic protein (GFAP), synaptophysin, and Ki-67 protein. Histologically, the tumour consisted of morphologically and immunohistochemically distinct components. Small pseudopapillary structures were seen consisting of hyalinized small vessels (Figure 10), which were covered by GFAP-positive pseudo stratified glial cells with small round nuclei (Figure 11).

**Figure 12:**
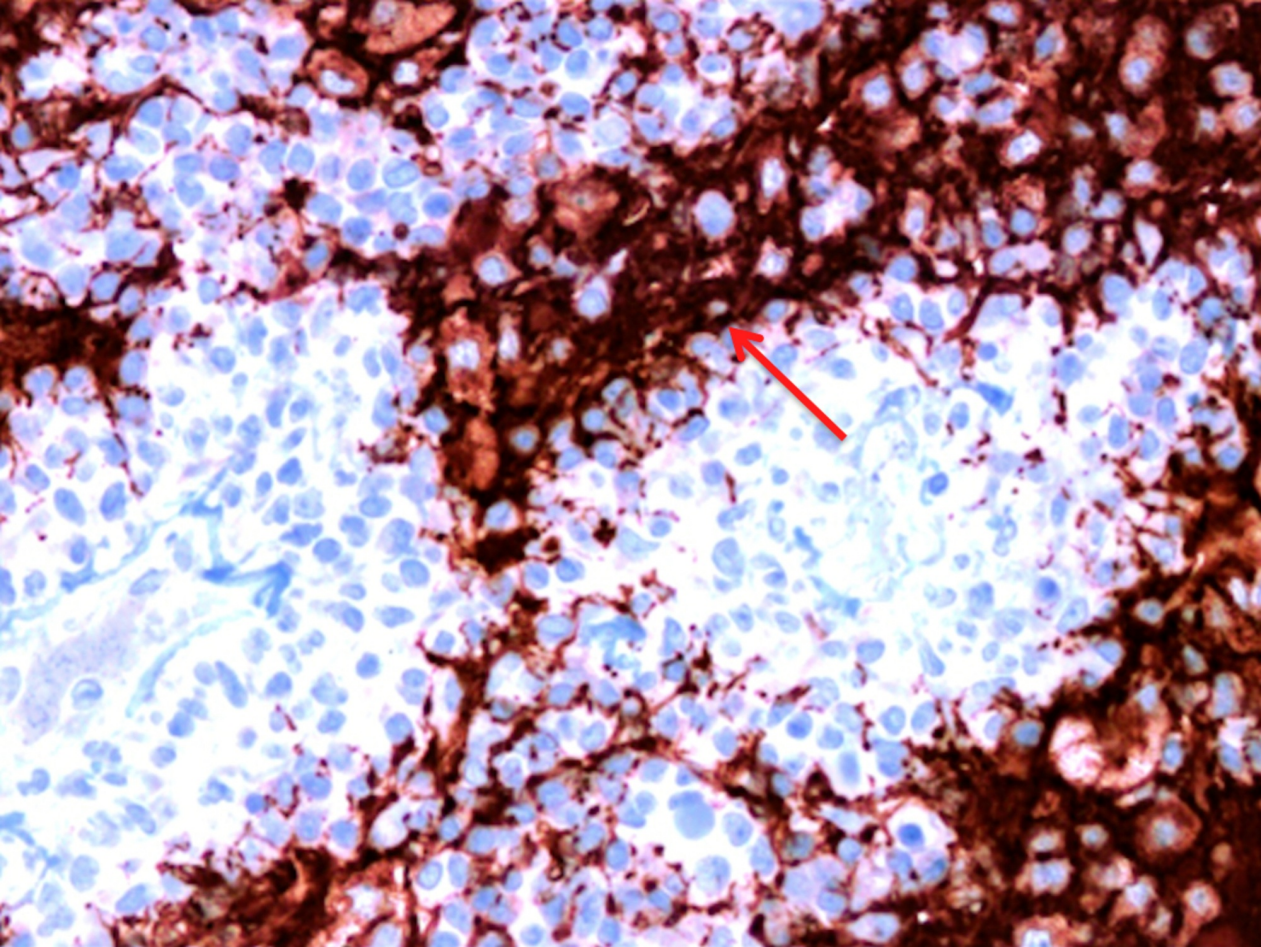
A 40x magnification view of small GFAP-positive cells surrounding vessels (arrows). GFAP: Glial fibrillary acidic protein.

The interpapillary space contained cells varying in size from small neurocyte cells to large ganglioid cells (some of them with neuronal nuclear features) that stained positive for synaptophysin (Figure 12).

**Figure 13:**
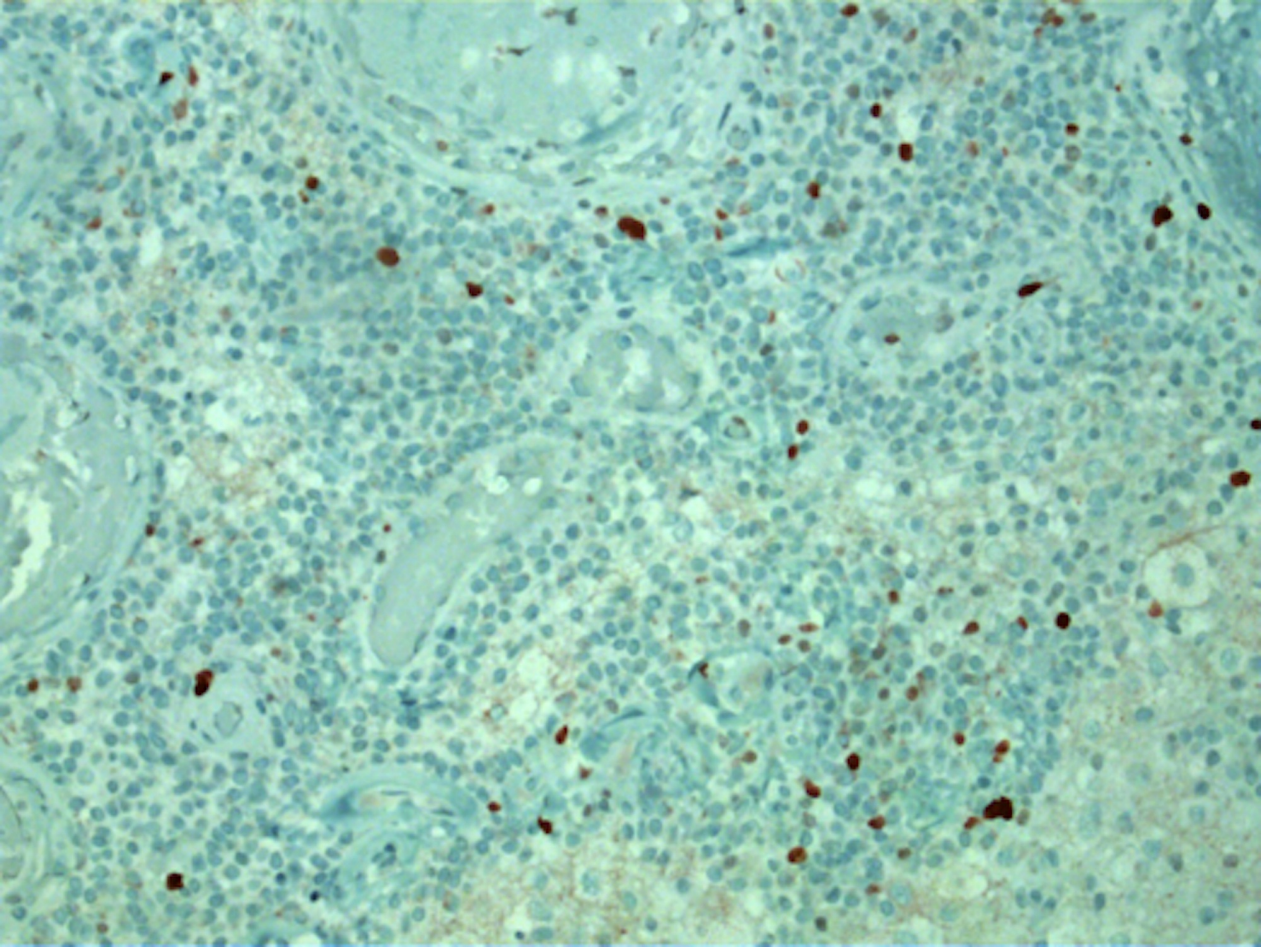
A 40x magnification view of low Ki-67 index.

The Ki-67 labeling index was low (Figure 13).

There were scattered hemosiderin deposits observed but no vascular proliferation or necrosis.

## Discussion

PGNT predominantly affects the younger population as the patients’ age varied from 11 to 41 years in the series reported by Li et al. [10]. The presenting symptoms of PGNT were related to increased intracranial pressure, seizures, focal neurological symptoms associated with tumour localization including hemiparesis, dysphasia, or visual disturbances [1, 6, 8-12].

Neuroimaging usually shows a cyst lesion with the most frequent pattern being a cystic and solid tumour or cystic tumour with mural nodule [1, 9, 13]. Purely cystic and solid tumour cases were less common [10, 14]. Cystic components are often hypointense on T1WI and hyperintense or isointensive on T2WI views. The patterns of contrast accumulation vary from heterogeneous to homogeneous, or ring enhancement, but, in most cases, the solid parts of tumours enhance with gadolinium. The majority of tumours are located supratentorially and it is usually stated that PGNT has an affinity for the frontal lobe, followed by the temporal, and parietal lobe involvement [15]. Although a few intraventricular cases are reported in the literature, PGNTs are usually detected in close proximity to the ventricles [15]. This is presumed to be related to a possible derivation of the tumour from the subependymal plate, which is known to produce bipotential neuroglial progenitor cells and some of them might persist in the region of the lateral ventricles in the brain [1].

Except for a few reported cases of aggressive behavior [1, 5, 7], histologically PGNT is a low-grade tumour, a member of a mixed neuronal-glial tumour family with its distinct pseudopapillary architecture. It is a biphasic tumour with papillary and solid parts, consisting of neurocytic and glial components. In our case, the tumour displayed typical histological and immunochemical features, but oligodendroglial-like cells and minigemistocytes may also be present [6-9, 13].

The patients diagnosed with PGNT usually undergo gross-total or partial resections, although radiotherapy and chemotherapy for more aggressive tumours were also reported [7, 9-11]. The extent of resection is a strong prognostic factor [16] and, although recurrences are infrequent, they are often associated with partial resections [6, 8, 9, 11, 17] and potentially the location of tumour in the parietal lobe [8, 9]. This could be related to an increased probability of postoperative defects (proximity of the eloquent cortex, internal capsule, and basal ganglia) and not the more aggressive nature of these tumours. In our case, the intraoperative pathology revealed no tumour in the walls of the cyst, so the decision was made only to remove the nodule and leave the cyst intact. The absence of postoperative defects and clinical outcome so far support this decision, although a long-term follow-up is necessary.

The case presented here is rare, as only a few patients with predominantly focal neurological deficits were reported in the literature so far [8-10]. Our patient, despite a visible mass effect on MRI and focal neurologic symptomatology, denied signs of raised intracranial pressure, which could be associated with a slowly progressing disease. The increasing number of reported cases and longer follow-up periods provide us with more insight into behavior and prognosis of this tumour.

## Conclusions

Patients with supratentorial, paraventricular cystic lesions, and intracystic septal enhancements should be suspected of having PGNT. The tumour is benign and gross-total resection provides the cure for patients. In the selected cases, a favorable outcome could be achieved by removing only the nodular component, however, a long-term follow-up is important to confirm the true biological behavior of PGNT.
